# Calgizzarin (S100A11): a novel inflammatory mediator associated with disease activity of rheumatoid arthritis

**DOI:** 10.1186/s13075-017-1288-y

**Published:** 2017-04-26

**Authors:** Lucie Andrés Cerezo, Barbora Šumová, Klára Prajzlerová, David Veigl, Dres Damgaard, Claus Henrik Nielsen, Karel Pavelka, Jiří Vencovský, Ladislav Šenolt

**Affiliations:** 1 Institute of Rheumatology, Na Slupi 4, 12850 Prague, Czech Republic; 2 Department of Rheumatology, 1st Faculty of Medicine, Charles University, Prague, Czech Republic; 3 First Orthopaedic Clinic, 1st Faculty of Medicine, Charles University, Prague, Czech Republic; 4 Institute for Inflammation Research, Center for Rheumatology and Spine Diseases, Copenhagen University Hospital, Rigshospitalet, Copenhagen, Denmark

**Keywords:** Calgizzarin, S100 proteins, Rheumatoid arthritis, Inflammation, Disease activity

## Abstract

**Background:**

Calgizzarin (S100A11) is a member of the S100 protein family that acts in different tumors by regulating a number of biologic functions. Recent data suggest its association with low-grade inflammation in osteoarthritis (OA). The aim of our study is to compare S100A11 expression in the synovial tissues, synovial fluid and serum of patients with rheumatoid arthritis (RA) and osteoarthritis (OA) and to characterize the potential association between S100A11 and disease activity.

**Methods:**

S100A11 protein expression was detected in synovial tissue from patients with RA (*n* = 6) and patients with OA (*n* = 6) by immunohistochemistry and immunofluorescence. Serum and synovial fluid S100A11 levels were measured by ELISA in patients with RA (*n* = 40) and patients with OA (*n* = 34). Disease activity scores in 28 joints based on C-reactive protein (DAS28-CRP) were used to assess disease activity. Cytokine content in peripheral blood mononuclear cells (PBMCs), synovial fibroblasts (SFs) and synovial fluid was analysed by ELISA, western blotting or cytometric bead array.

**Results:**

S100A11 expression was significantly up-regulated in the synovial lining and sublining layers (*p* < 0.01) and vessels (*p* < 0.05) of patients with RA compared to patients with OA, and was associated with fibroblasts and T cells. S100A11 was significantly increased in synovial fluid (*p* < 0.0001) but not in serum (*p* = 0.158) from patients with RA compared to patients with OA when adjusted for age and sex. Synovial fluid S100A11 correlated with DAS28 (*r* = 0.350, *p* = 0.027), serum CRP (*r* = 0.463, *p* = 0.003), synovial fluid leukocyte count (*r* = 0.677, *p* < 0.001), anti-cyclic citrullinated peptide antibodies (anti-CCP) (*r* = 0.424, *p* = 0.006) and IL-6 (*r* = 0.578, *p* = 0.002) and IL-8 (*r* = 0.740, *p* < 0.001) in synovial fluid from patients with RA. PBMCs and SFs isolated from patients with RA synthesized and spontaneously secreted higher levels of S100A11 in comparison with PBMCs and SFs from patients with OA (*p* = 0.011 and 0.03, respectively). S100A11 stimulated the production of the pro-inflammatory cytokine IL-6 by PBMCs (*p* < 0.05) and SFs (*p* < 0.01).

**Conclusions:**

Our data provide the first evidence of S100A11 up-regulation and its association with inflammation and disease activity in patients with RA.

**Electronic supplementary material:**

The online version of this article (doi:10.1186/s13075-017-1288-y) contains supplementary material, which is available to authorized users.

## Background

Rheumatoid arthritis (RA) is a chronic systemic autoimmune disease with predominant musculoskeletal manifestations. The hallmark of this condition is persistent synovial inflammation leading to irreversible structural damage and joint failure. Despite intensive research, the exact cause of RA remains elusive. It is well-established that activated immune cells and synovial fibroblasts producing inflammatory factors play a central role in the pathophysiologic mechanisms of RA [1]. Nevertheless, clarifying the roles of cytokines within a complex regulatory network in RA remains a key challenge.

S100A11 (also known as S100C or calgizzarin) is a less well-known member of the large calcium-binding S100 protein family [2] that has been proposed to play specific biologic roles in the processes of endocytosis and exocytosis [3–5], enzyme activity regulation [6], cell growth [7], apoptosis [8] and low-grade inflammation [9]. S100A11 is expressed at different levels in various tissues [10] and is localized in the nucleus, cytoplasm and cell periphery [11–13]. S100A11 is associated with the oncogenesis of many different types of tumour and has been identified as a tumour suppressor in some cancers and as a tumour promoter in other cancers [14]. S100A11 has been found to play an ambivalent role in growth regulation [11, 15] and acts via receptor for advanced glycation end-products (RAGE)-dependent signalling in human keratinocytes [11]. Moreover, S100A11 has been shown to regulate the stability of the cell cycle regulator, cyclin-dependent kinase inhibitor (1p21CIP1/WAF1) in human keratinocytes [16].

Increased S100A11 expression was recently demonstrated in human articular cartilage in patients with osteoarthritis (OA) [17, 18]. S100A11 expression and release from chondrocytes were induced by the pro-inflammatory cytokines interleukin-1β (IL-1β) and tumour necrosis factor-α (TNF-α) and the chemokine CXCL8 [9, 18]. Moreover, extracellular S100A11 was shown to act via RAGE-dependent signalling to activate the p38 mitogen-activated protein kinase (MAPK) pathway and accelerate chondrocyte hypertrophy and matrix catabolism [18]. In addition, S100A11 and several other S100 proteins are up-regulated in the early preclinical phase (before signs of arthritis are present) of adjuvant arthritis [19].

Therefore, the aim of our study was to assess local and systemic S100A11 expression in patients with RA and OA and to investigate the relationship among S100A11, inflammation and RA disease activity.

## Methods

### Patients

We included 40 patients with active RA (27 female and 13 male, mean age ± SD 54.90 ± 14.25) and 34 patients with knee OA (25 female and 9 male, mean age ± SD 65.20 ± 10.41); see Table 1 for patient characteristics. Patients with active RA met the American College of Rheumatology criteria for the diagnosis of RA [20]. This study had approval from the Ethics Board of the Institute of Rheumatology, and all subjects provided informed written consent to participate in it.

**Table 1:** Characteristics of patients with rheumatoid arthritis (RA) and osteoarthritis (OA)

Characteristics	RA (*n* = 40)	OA (*n* = 34)
Gender (female/male)	27/13	25/9
Age (years)	54.90 ± 14.25	65.21 ± 10.41
CRP (mg/l)	27.88 ± 31.80	4.11 ± 4.30
Disease duration (years)	6.72 ± 8.58	5.57 ± 7.41
Drugs (DMARDs/GCs)	37/26	-
Biological therapy	8^a^	-
DAS28	4.39 ± 1.19	-
RF positivity, *n* (%)	31 (72%)	-
Anti-CCP positivity, *n* (%)	26 (60%)	-

Data are expressed as the mean (± SD) unless stated otherwise. ^a^Out of 8 patients, 5 were treated with anti-TNF therapy, 1 with tocilizumab, 1 with rituximab and 1 with anti-IL-17 therapy. *Anti-CCP* anti-cyclic citrullinated peptide antibody, *BMI* body mass index, *CRP* C-reactive protein, *DAS28* disease activity score in 28 joints, *DMARDs* disease-modifying antirheumatic drugs, *GCs* glucocorticoids, *MTX* methotrexate, *RF* rheumatoid factor, *SJC* swollen joints count

### Laboratory measurements

Paired samples of peripheral blood and synovial fluid were collected at the time of clinically indicated knee arthrocentesis, immediately processed, and stored at -80 °C until use. Prior to analysis, the synovial fluid samples were treated with hyaluronidase (Hylase Dessau; Riemser Arzneimittel, Greifswald, Germany) for 30 minutes at 37 °C. The clinical assessments were performed using the disease activity score in 28 joints based on C-reactive protein (DAS29-CRP). CRP was measured in serum by turbidimetry using an Olympus Biochemical Analyzer (Olympus CO Ltd., Tokyo, Japan). Serum anti-cyclic citrullinated peptide antibody (anti-CCP) and IgM rheumatoid factor (IgM-RF) levels were determined using standard ELISA kits (Test Line s.r.o., Czech Republic).

### Measurement of S100A11 and cytokines

Serum and synovial fluid S100A11 levels were measured using a commercially available ELISA kit, according to the manufacturer’s protocol (BioVendor, Brno, Czech Republic). The detection limit of the assay is 0.01 ng/ml. The protein levels of the following cytokines in the cell culture supernatants were analysed using the indicated ELISA kits, according to the manufacturers’ protocols: TNF-α and IL-6 (Ray Biotech, Norcross, GA, USA) and IL-1β and monocyte chemotactic protein 1 (MCP-1) (R&D Systems, Wiesbaden-Nordenstadt, Germany). The analyses were performed using a SUNRISE ELISA reader (Tecan, Salzburg, Austria) at 450 nm.

The BD Cytometric Bead Array Human Inflammatory Cytokine Kit II (Catalogue number 551811, BD Biosciences, Franklin Lakes, NJ, USA) was used to measure IL-1β, IL-6, IL-8, IL-10 and TNF-α in 5 μl synovial fluid diluted in 20 μl PBS as described in a previous study [21], which included 26 of the patients with RA and 21 of the patients with OA examined in the present study.

### Cell isolation, cell cultures and stimulation experiments

Peripheral blood mononuclear cells (PBMCs) were purified using standard Ficoll-Paque density gradient centrifugation. The PBMCs were seeded in 6-well culture plates at a density of 1.0 × 10^6^ cells (to study spontaneous release of S100A11) or 5.0 × 10^5^ cells (for stimulation experiments) per 1 ml of Advanced RPMI-1640 culture medium (Invitrogen, Carlsbad, CA, USA) and were incubated in a humidified atmosphere for 30 minutes at 37 °C with 5% CO_2_. For S100A11 analysis, cells from patients with RA (*n* = 9) and OA (*n* = 9) were incubated for 24 h without treatment. The cell culture supernatants were collected and stored at -80 °C.

Synovial fibroblasts (SFs) were isolated from biopsies from patients with RA (*n* = 6) and patients with OA (*n* = 6), which were obtained during surgery as previously described [22]. SFs (passages 4–6) were seeded at a density of 1.0 × 10^5^ cells/per well in 6-well culture plates in 1 ml of DMEM without serum. After the cells were incubated for 24 h without treatment, their supernatants were collected and stored at -80 °C.

PBMCs (n = 8) and SFs (n = 8) from patients with RA (both obtained as described previously) were treated with recombinant TNF-α (10 ng/ml), IL1-β (10 ng/ml), IL-6 (10 ng/ml) (all from R&D Systems, Minneapolis, MN, USA), lipopolysaccharide (LPS) (10 ng/ml) (Sigma-Aldrich, St Louis, MO, USA), polyinosinic:polycytidylic acid (Poly (I:C)) (20 μg/ml) (Invivogen, San Diego, CA, USA) or different concentrations (100–1000 ng/ml) of recombinant S100A11 protein (Biovendor, Brno, Czech Republic). The doses of recombinant S100A11 were selected based on the concentration range detected in the serum and synovial fluid of patients with RA. After the cells were incubated for 24 h, their supernatants were collected and stored at -80 °C.

### Immunohistochemical assessment

Samples from synovial biopsies were obtained from patients with RA (four female and two male, mean age ± SD 54.83 ± 9.41 years) from one hip joint, two knee joints, two elbow joints and one hand joint and from six patients with OA (three female and three male, mean age ± SD 66.00 ± 7.77 years) from four knee joints and two hip joints during joint surgery; the samples comprised 5-mm-thick paraffin-embedded sections. The sections were deparaffinised and rehydrated, and endogenous peroxidase activity and background binding were inhibited using 3% hydrogen peroxide in methanol and 1% bovine serum, respectively. Synovial tissue sections were processed for antigen retrieval and immunohistochemical assessment as described elsewhere [23]. The slides were immunoprobed with polyclonal rabbit S100A11 antibodies (ProteinTech, Chicago, IL, USA) in a dilution of 1:20. Blinded, semiquantitative evaluation was performed using a BX53 microscope and DP80 Digital Microscope Camera and CellSens Standard Software (Olympus, Philadelphia, PA, USA). Eight to ten random and non-overlapping fields of synovial tissue were analysed, and S100A11 staining intensity was scored according to a 4-point scale, in which a score of 0 represented negative staining, and scores of 1–3 represented weak, moderate and strong staining intensity, respectively.

### Immunofluorescence

For the double-staining experiments, formalin-fixed, paraffin-embedded synovial sections from patients with RA (*n* = 4) were incubated with primary antibodies against S100A11 (diluted 1:100; Abcam, Cambridge, UK), CD68 (diluted 1:100; Abcam, Cambridge, UK), CD3 (diluted 1:100; Abcam, Cambridge, UK), vimentin (diluted 1:50; Sigma-Aldrich, St. Louis, MO, USA), and CD20 (diluted 1:50; Abcam, Cambridge, UK) before being incubated with secondary antibodies coupled to Alexa 488 or Alexa 647 (diluted 1:200) (Abcam, Cambridge, UK). The samples were then analysed using a BX53 microscope with a DP80 Digital Microscope Camera and CellSens Standard Software (Olympus, Philadelphia, PA, USA). S100A11 positivity in different cell populations was quantified in six randomly selected high-power fields per patient by two experienced researchers in a blinded manner and expressed as percentage of S100A11-positive cells.

### Western blot analysis

PBMCs and synovial fibroblasts were homogenized and lysed in radioimmunoprecipitation assay (RIPA) buffer (Thermo Fisher, Waltham, MA, USA). Proteins were separated by tricine sodium dodecyl sulfate-polyacrylamide gel electrophoresis and transferred to polyvinylidene difluoride membranes (Biorad Hercules, CA, USA), which were incubated with antibodies against S100A11 (1:100) (Abcam, Cambridge, UK) overnight at 4 °C. Horseradish-peroxidase-conjugated antibodies (1:2000) (Dako, Glostrup, Denmark) were used as secondary antibodies. Beta-actin antibodies (1:5000) (Abcam, Cambridge, UK) were used as loading controls. The ECL™ Prime Western Blotting System (Sigma-Aldrich, St. Louis, MO, USA) was used for protein visualization. The signals were recorded by an Azure Biosystems c300 imaging system (Azure Biosystems, Dublin, CA, USA).

### Statistical analysis

The Kolmogorov-Smirnov test of normality was used for all variables. S100A11 in serum and synovial fluid were expressed as median (range). Differences in S100A11 levels between the two groups were analysed using the Mann-Whitney *U* test. Analyses of differences between the groups were further adjusted for confounders (sex and age) using a generalized linear model. The relationships between non-normally distributed variables were determined by calculating the Spearman correlation coefficient. Differences in synovial tissue or PBMC S100A11 expression levels between patients with RA and OA were determined using the Mann-Whitney *U* test. The unpaired *t* test was used for analysis of protein synthesis. Data are expressed as the mean (SEM) unless stated otherwise. *P* values below 0.05 were considered statistically significant. GraphPad Prism 5 (GraphPad Software, San Diego, CA, USA) or R (R Foundation for Statistical Computing, Vienna, Austria) was used to perform the analysis.

## Results

### S100A11 is up-regulated in rheumatoid arthritis synovial tissue

**Fig. 1:**
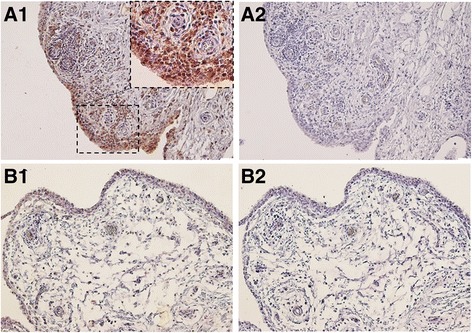
S100A11 protein in rheumatoid arthritis (RA) and osteoarthritis (OA) synovial tissue assessed by immunohistochemical (IHC) analysis. Intensive staining for S100A11 was found in the synovial fibroblasts of the lining layer and within the inflammatory cell infiltrates in the RA synovial tissue (*A1*). The S100A11 expression was negligible or absent in OA synovium (*B1*). Mouse IgG was used as an isotype control (*A2*, *B2*). Representative images of IHC staining are shown at × 200 magnification. For the detailed view the × 400 magnification is shown (*n* = 6 patients with RA and *n* = 6 patients with OA)

S100A11 protein was up-regulated in synovial tissue from patients with RA compared to synovial tissue from patients with OA, in which rather negligible S100A11 expression was observed (Fig. 1). Expression of S100A11 was markedly increased in the cells of the synovial lining layers (*p* < 0.01), interstitium (*p* < 0.01) and inflammatory infiltrates (*p* < 0.01) in samples from patients with RA compared to patients with OA. S100A11 expression in synovial tissue vessels was slightly higher in samples from patients with RA than from patients with OA (*p* < 0.05) (Table 2).

**Table 2:** Expression of S100A11 related to different cellular compartments in synovial tissues from patients with rheumatoid arthritis (RA) and osteoarthritis (OA)

	RA	OA	Mann-Whitney test
Lining layer	2.58 ± 0.20	1.00 ± 0.29	*p* < 0.01
Interstitium	2.25 ± 0.11	0.25 ± 0.11	*p* < 0.01
Inflammatory infiltrate	2.42 ± 0.27	0.50 ± 0.18	*p* < 0.01
Vessels and capillaries	1.33 ± 0.17	0.33 ± 0.21	*p* < 0.05

The intensity of S100A11 expression was scored using a semiquantitative 4-point scale. A score of 0 represented no staining, 1 weak, 2 moderate and 3 strong staining intensity. Values represent mean (± SD)

**Fig. 2:**
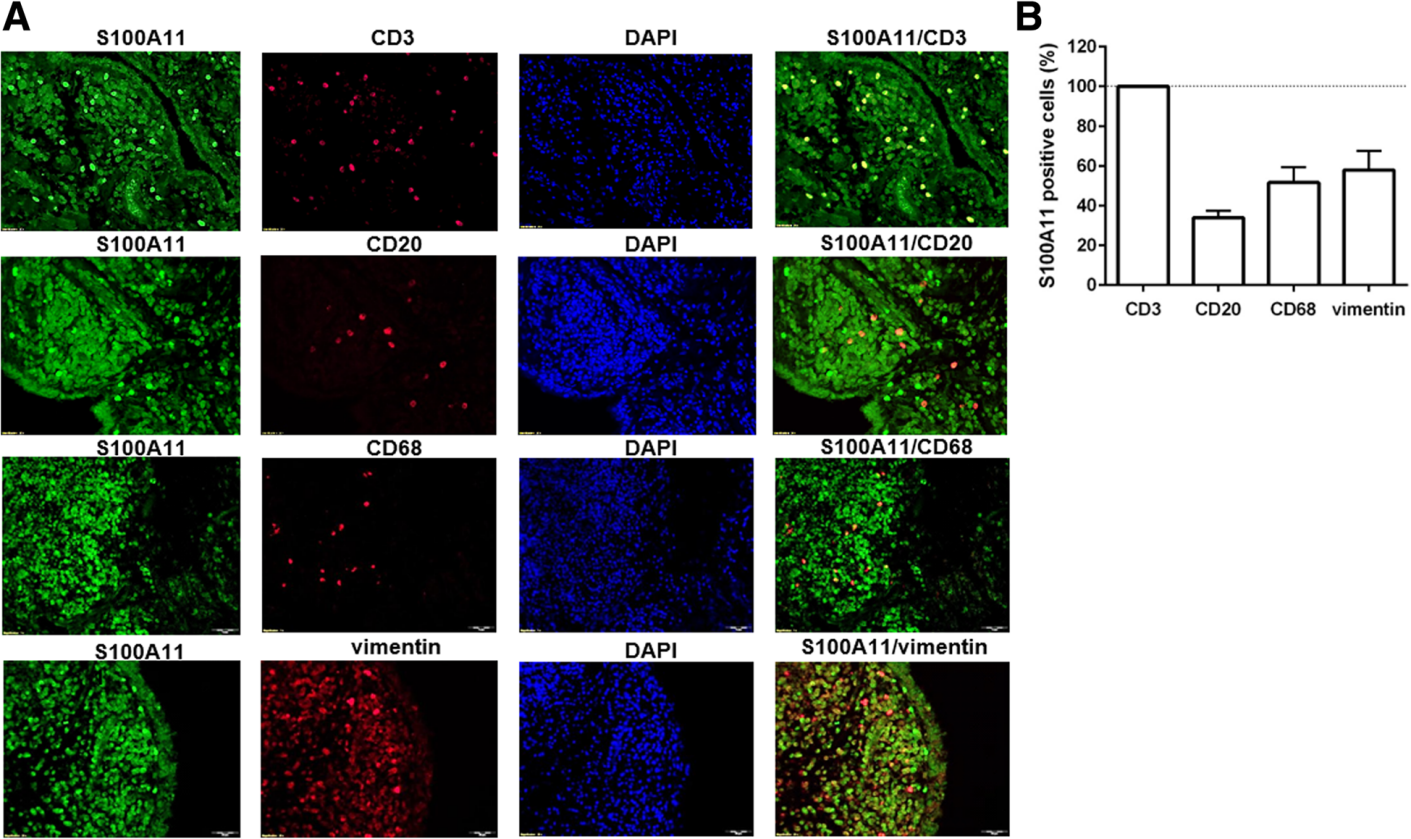
Cellular distribution of the S100A11 protein in rheumatoid arthritis synovial tissue. Representative images of immunofluorescence staining (**a**) for CD68 (macrophages), CD20 (B cells), CD3 (T cells) and vimentin (cells of mesenchymal origin) are shown at × 200 magnification (*n* = 4). *DAPI* 4',6-diamidino-2-phenylindole. **b** Average percentage of S100A11-positive cells presented as mean ± SEM

To determine which synovial cells express S100A11, we preformed double-immunofluorescence staining using fibroblast (vimentin), macrophage (CD68), B cell (CD20) and T cell (CD3) markers. We observed significant vimentin and S100A11 co-expression, indicating that the majority of S100A11-positive cells were synovial fibroblasts. We also found that S100A11 was expressed by T cells, some macrophages and only some B cells (Fig. 2a, b).

### Serum and synovial fluid S100A11 levels are increased in rheumatoid arthritis

**Fig. 3:**
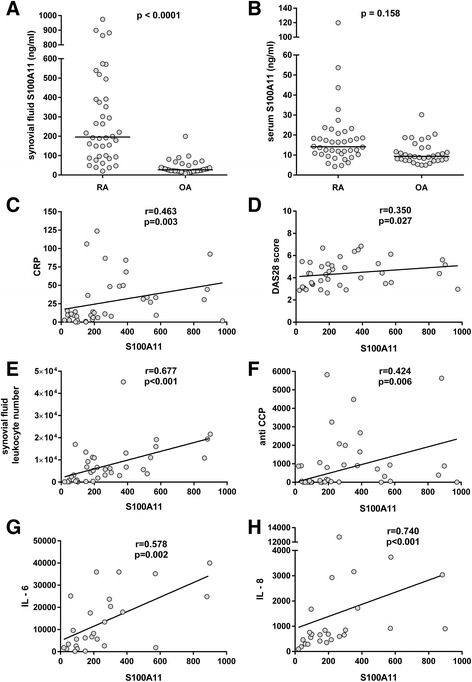
S100A11 in serum and synovial fluid and its association with disease activity in rheumatoid arthritis (*RA*). Levels of S100A11 in the synovial fluid (**a**) but not in the serum (**b**) were up-regulated in patients with RA compared to patients with osteoarthritis (*OA*) when adjusted for age and sex. Levels of S100A11 in synovial fluid correlated with serum C-reactive protein (*CRP*) (**c**), disease activity score in 28 joints (*DAS28*) (**d**), synovial fluid leukocyte count (**e**), serum anti-cyclic citrullinated peptide antibodies (*anti-CCP*) (**f**), IL-6 (**g**) and IL-8 (**h**). The *horizontal line* represents the median

Levels of S100A11 in the synovial fluid (195.8 (20.2–974.2) vs. 26.8 (8.2–199.4) ng/ml; *p* < 0.0001) but not in the serum (14.1 (4.3–119.8) vs. 9.3 (5.0–30.2) ng/ml; *p* = 0.158) were significantly up-regulated in patients with RA compared to patients with OA when adjusted for age and sex (Fig. 3a, b). S100A11 levels in serum or synovial fluid were not affected by treatment. Synovial fluid S100A11 levels were significantly higher than systemic S100A11 levels in both RA (*p* < 0.0001) and OA (*p* < 0.0001), but there was no association between serum and synovial fluid S100A11 levels in either group.

The levels of S100A11 did not differ between double-positive (RF+/anti-CCP+) and double-negative (RF-/anti-CCP-) patients with RA in the serum or synovial fluid. Significant up-regulation of S100A11 levels in the synovial fluid but not in the serum was observed in anti-CCP-positive compared to anti-CCP-negative patients (*p* = 0.007). The levels of S100A11 did not differ between RF-positive and RF-negative patients with RA (Additional file 1).

### Synovial fluid S100A11 levels are associated with inflammatory markers and with rheumatoid arthritis disease activity

In RA patients, synovial fluid S100A11 levels were significantly associated with disease activity as represented by the DAS28 (*r* = 0.350, *p* = 0.027), CRP (*r* = 0.463, *p* = 0.003) and synovial fluid leukocyte count (*r* = 0.677, *p* < 0.001) (Fig. 3c–e). Furthermore, synovial fluid S100A11 levels were correlated with serum anti-CCP levels (*r* = 0.424, *p* = 0.006) (Fig. 3f), but not IgM-RF levels (*r* = 0.059, *p* = 0.719), in patients with RA. Correlation between S100A11 and CRP (*r* = 0.458, *p* = 0.004), DAS28 (*r* = 0.414, *p* = 0.011) or synovial fluid cell count (*r* = 0.703, *p* < 0.001) was not affected by age, BMI or anti-CCP levels. As there was no association between serum and synovial fluid S100A11 levels, serum S100A11 levels were not correlated with any clinical or laboratory RA disease activity parameters.

We further analysed the selected cytokines in the synovial fluid of patients with RA and OA (Additional file 2) and their association with the levels of S100A11. Synovial fluid S100A11 was significantly correlated with the levels of IL-6 (*r* = 0.578, *p* = 0.002) and IL-8 (*r* = 0.740, *p* < 0.001) (Fig. 3g, h), but not with the levels of IL-1β, TNF-α or IL-10 in patients with RA. In patients with OA, significant association was only found between S100A11 and IL-8 in the synovial fluid (*r* = 0.592, *p* = 0.005).

### Peripheral blood mononuclear cells and synovial fibroblasts from patients with rheumatoid arthritis synthesize and spontaneously release higher levels of S100A11

**Fig. 4:**
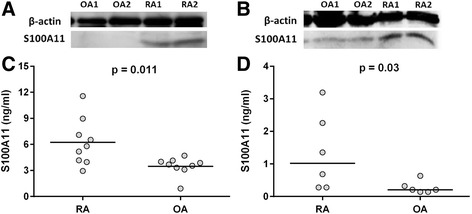
Synthesis and release of S100A11 by peripheral blood mononuclear cells (PBMCs) and synovial fibroblasts (SFs). PBMCs and SFs from patients with rheumatoid arthritis (*RA*) synthesize (**a**, **b**) and spontaneously release (**c, d**) higher levels of S100A11 compared to the cells from patients with osteoarthritis (*OA*). Representative images of protein levels of S100A11 in PBMCs and SFs isolated from patients with RA (*n* = 4) and patients with OA (*n* = 4) by western blot

To identify potential S100A11 sources in patients with RA, we analysed S100A11 protein synthesis and release from selected cells. As shown by western blotting (Fig. 4a, b), S100A11 synthesis was up-regulated in PBMCs and SFs in patients with RA compared to the PBMCs and SFs in patients with OA. Consistent with these findings, we showed that PBMCs (3.12 ± 0.45 vs. 1.74 ± 0.18 ng/ml, *p* = 0.011) and SFs (1.34 ± 0.48 vs. 0.30 ± 0.06 ng/ml, *p* = 0.054) in patients with RA spontaneously released larger amounts of S100A11 into the cell culture supernatants than the cells did in patients with OA (Fig. 4c, d). Neither pro-inflammatory cytokines (IL-1β, IL-6 or TNF-α) nor LPS up-regulated the production of S100A11 by PBMCs or SFs above the spontaneous release (Additional file 3A, B). We have observed enhanced S100A11 release from SFs, but not PBMCs, upon poly I:C treatment (Additional file 3B).

### S100A11 enhances pro-inflammatory cytokine release by peripheral blood mononuclear cells and synovial fibroblasts

Given the observed increases in S100A11 accumulation in patients with RA, we investigated the role of extracellular S100A11 in inflammation. PBMCs and SFs isolated from patients with RA were exposed to recombinant S100A11 for 24 h, and TNF-α, IL-1β, IL-6 and MCP-1 levels in the cell culture supernatants were analysed. S100A11 concentrations were selected based on the mean serum and synovial fluid protein levels of patients with OA or RA.

**Fig. 5:**
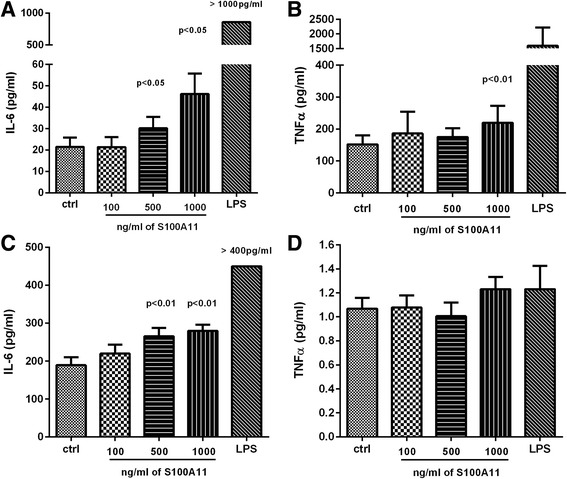
Pro-inflammatory role of extracellular S100A11. S100A11 significantly enhances the release of IL-6 (**a**) and TNF-α (**b**) in peripheral blood mononuclear cells and IL-6 (**c**) in synovial fibroblasts (SFs). Levels of TNF-α were not changed in SFs treated with S100A11 protein (**d**). Protein levels in cell culture supernatants were measured after 24 h. The *horizontal line* represents the median. *Ctrl* control, *LPS* lipopolysaccharide

Exposure to recombinant S100A11 protein enhanced IL-6 release into the cell supernatant in a dose-dependent manner in PBMCs (1000 ng/ml: 33.51 (26.46–95.55), *p* < 0.05; 500 ng/ml: 25.63 (18.16–54.51), *p* < 0.05; 100 μg/ml: 16.92 (12.35–47.64), compared to unstimulated: 15.26 (9.82–39.76)) and synovial fibroblasts (1000 ng/ml: 281.6 (219.7–363.6), *p* < 0.01; 500 ng/ml: 245.3 (196.5–355.4), *p* < 0.01; 100 μg/ml: 195.2 (148.6–328.0), compared to unstimulated: 193.0 (90.08–279.0)) (Fig. 5a, b).

TNF-α production was significantly increased by exposure to higher S100A11 doses in PBMCs (1000 ng/ml: 206.5 (161.8–326.4) vs. unstimulated: 153.4 (110.7–190.0), *p* < 0.01), but not SFs (Fig. 5c, d). MCP-1 and IL-1β levels were not affected by S100A11 treatment in PBMCs or SFs (data not shown).

## Discussion

In the present study, we demonstrated for the first time the up-regulation of S100A11 in patients with RA and the association between S100A11, inflammation and disease activity. Numerous studies have reported that specific members of the S100 protein family participate in immune response activation and inflammation in RA [24–27]; however, very little is known about the involvement of S100A11 in RA. The only indication of a link between S100A11 and RA was noted by Yu et al. [19], who described a rat model of adjuvant arthritis showing increased S100A11 expression during the early preclinical phase of experimental arthritis. Associations between S100A11 and low-grade inflammation and cartilage destruction have been previously described in OA [9, 18].

Here, we reported significant S100A11 accumulation in the synovial tissues of patients with RA, particularly in the synovial lining layer and inflammatory infiltrates. This finding corresponds with those of previous studies documenting the topography of other S100 proteins associated with RA [22, 24, 25]. Similar to S100A4 [22], S100A11 is strongly expressed by T cells and SFs, which therefore represent the main extracellular sources of S100A11 for the joint environment.

Consistent with the accumulation of S100A11 in RA synovial tissue was our finding of significantly elevated S100A11 expression in the synovial fluid and serum of patients with RA. The up-regulation of S100A11 in serum from patients with RA was somewhat less pronounced than the up-regulation of S100A11 in synovial fluid from patients with RA and was not significant when adjusted to age and sex. In addition, we observed no changes in serum S100A11 levels in patients with recent-onset RA compared to healthy controls (unpublished data). These findings imply that the joint microenvironment is a major source of S100A11 and that S100A11 could diffuse from the inflamed RA synovium into the circulation.

Interestingly, the serum and synovial fluid levels of S100A11 were markedly lower than the corresponding levels of other S100 family members involved in RA [22, 24, 25]. Nonetheless, synovial fluid S100A11 is significantly related to clinical disease activity in RA, reflecting a local, ongoing inflammatory process. Moreover, given the strong correlation between synovial fluid S100A11 levels and synovial fluid leukocyte count, we can speculate that the S100A11 levels may also be associated with the amount of immune cells within the inflamed synovial tissue. Accompanied by the correlation between synovial fluid S100A11 and the inflammatory status and with the synovial fluid cytokines (IL-6 and IL-8), these data further strengthen the hypothesis of S100A11 involvement in the local inflammatory process in RA. Indeed, S100A11 is significantly associated with IL-8 in synovial fluid in patients with OA. This is consistent with the up-regulation of S100A11 by human articular chondrocytes exposed to IL-8 and with its suggested role in low-grade local inflammation in OA (9). We also demonstrated association between S100A11 and anti-CCP autoantibodies and thereby with autoimmune response in RA, which is further supported by our observation of significantly higher levels of S100A11 in the synovial fluid of anti-CCP-positive compared to anti-CCP-negative patients.

Taken together, unlike other S100 proteins [24, 28–32], systemic levels of S100A11 cannot be considered a biomarker of disease activity. However the local up-regulation of S100A11 in the synovial compartment could reflect the inflammatory process and immune response in patients with RA.

In addition, we demonstrated increased S100A11 synthesis and spontaneous release by PBMCs and SFs in patients with RA. Active protein secretion has been described previously in chondrocytes [9] and keratinocytes [11]. However, in contrast to chondrocytes [9], treatment with pro-inflammatory cytokines does not up-regulate the production of S100A11 by PBMCs or SFs above the spontaneous release. This may be partly due to the lower reactivity of the chronically activated cells from patients with RA to an additional pro-inflammatory stimulus. However, in SFs exposed to the toll-like receptor 3 (TLR3) ligand poly (I:C) there were threefold increases in S100A11 production. As previously shown by Brentano et al., poly (I:C) and necrotic synovial fluid cells stimulate pro-inflammatory reactions in RA SFs via TLR3 [33].

It is likely that in RA, local destructive events in the synovial joints accompanied by the release of endogenous double stranded RNA (dsRNA) from necrotic cells can lead to the activation of tissue-resident SFs to release S100A11. On the other hand, our recent findings indicate no direct effect of S100A11 on the expression of matrix-degrading enzymes by SFs and thereby on joint destruction (data not shown). Moreover, by investigating the potential role of S100A11 as an extracellular regulatory molecule, we found that recombinant S100A11 when used in the same concentrations as in synovial fluid acts on both SFs and PBMCs to enhance IL-6 production and thus amplify inflammation. In line with that, synovial fluid levels of S100A11 correlated significantly with that of IL-6. However, it must be noted that the cytokine-like effects of S100A11 seem to be less potent than those of other S100 proteins involved in RA [26, 34]. Nevertheless, it is plausible that S100A11 in synovial fluid could enhance pro-inflammatory cytokine secretion to form a positive feedback loop in RA. The relative contributions of certain S100 proteins to the immune response in RA are different, and it is likely that S100A11 participates in several pathological processes in RA other than inflammation.

This study has some limitations. First, due to the cross-sectional study design, a longitudinal causal relationship between the modulation of S100A11 levels and changes in disease activity in time cannot be assessed. Moreover, a relatively small number of patients were enrolled in our study. Therefore, a long-term, large-scale, prospective study is warranted to further investigate the role of S100A11 in RA. Second, the relationship between histopathological scoring of levels of S100A11 in the synovium and synovial fluid could not be assessed because synovial tissue was collected from a different group of patients to those who underwent aspiration of a knee joint effusion. Similarly, the relationship between destruction in the joint at the sampling site and S100A11 levels in synovial fluid was not described because radiographic evaluation of affected knee was not available in these subjects. Third, we identified significant differences in the synthesis and release of S100A11 between RA and OA cells; however, in this study we have not focused on the determination of different conformational forms of S100A11 and their potential relationship with inflammation. Last, as other members of the S100 protein family are known to be involved in the pathogenesis of RA, further studies on their relationship and potential co-regulation with S100A11 would be extremely interesting. In spite of these limitations, this study provides novel data showing a link between S100A11 and inflammation in RA.

## Conclusions

Taken together, our findings have provided us with novel insights into the involvement of S100A11 in the process of inflammation and pathogenesis of RA; however, its roles in the processes of joint inflammation and destruction seem to be complex and require further investigation.

## Additional files

Additional file 1: The levels of S100A11 protein in serum and synovial fluid of seropositive and seronegative patients with RA. Values are presented as mean ± SD. *P* values were determined using the Mann-Whitney test. (JPG 59 kb)

Additional file 2: The levels of selected cytokines in the synovial fluid of patients with RA and OA. (JPG 82 kb)

Additional file 3: The release of S100A11 protein remains unchanged in PBMCs (**A**) upon the treatment with pro-inflammatory cytokines, LPS or poly I:C. Synovial fibroblasts up-regulate the release of S100A11 when stimulated with poly I:C (**B**). Protein levels in cell culture supernatants were measured after 24 h. The *horizontal line* represents the median. (TIF 85 kb)

## Abbreviations

anti-CCP: Anti-cyclic citrullinated peptide antibodies
BMI: Body mass index
CRP: C-reactive protein
CXCL8: (C-X-C motif) ligand 8
DAS28: Disease activity score in 28 joints
DMARDs: disease-modifying antirheumatic drugs
DMEM: Dulbecco's modified Eagle’s medium
ELISA: Enzyme immunoassay
ESR: Erythrocyte sedimentation rate
EULAR: European League Against Rheumatism
IgM-RF: IgM rheumatoid factor
IL: Interleukin
LPS: Lipopolysaccharide
MAPK: Mitogen-activated protein kinase
MCP-1: Monocyte chemotactic protein 1
MTX: Methotrexate
OA: Osteoarthritis
p21Cip1/WAF1: Cyclin-dependent kinase inhibitor 1
PBMC: Peripheral blood mononuclear cell
PBS: Phosphate-buffered saline
PCR: Polymerase chain reaction
poly I:C: Polyinosinic:polycytidylic acid
RA: Rheumatoid arthritis
RAGE: Receptor for advanced glycation end products
RIPA: Radioimmunoprecipitation assay
SD: Standard deviation
SEM: Standard error of the mean
SF: Synovial fibroblast
SJC: Swollen joints count
TLR: Toll-like receptor
TNF: Tumour necrosis factor
