# Supplementary figures and images for: Calgizzarin (S100A11): a novel inflammatory mediator associated with disease activity of rheumatoid arthritis
Source: Arthritis Res Ther. 2017 Apr 26;19:79. doi: 10.1186/s13075-017-1288-y (PMC5405489; doi:10.1186/s13075-017-1288-y)

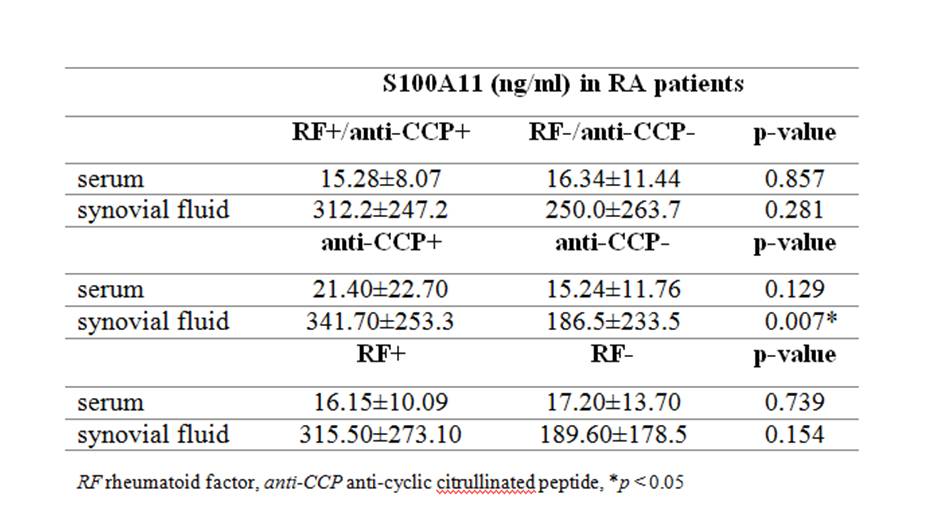

Supplement: Supplementary file 1 — The levels of S100A11 protein in serum and synovial fluid of seropositive and seronegative patients with RA. Values are presented as mean ± SD. P values were determined using the Mann-Whitney test. (JPG 59 kb) [file 13075_2017_1288_MOESM1_ESM.jpg]

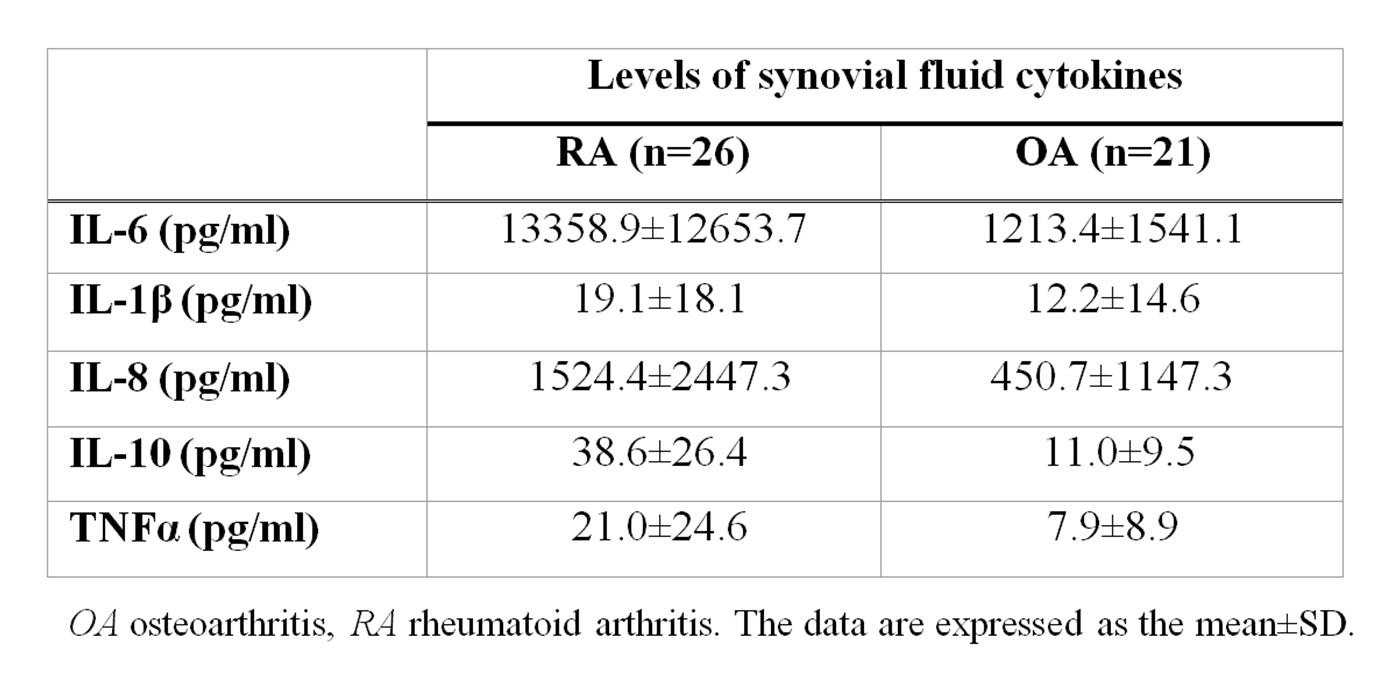

Supplement: Supplementary file 2 — The levels of selected cytokines in the synovial fluid of patients with RA and OA. (JPG 82 kb) [file 13075_2017_1288_MOESM2_ESM.jpg]

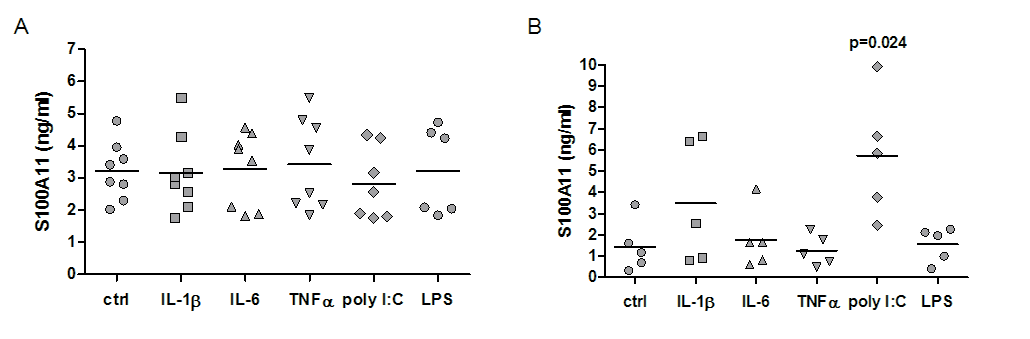

Supplement: Supplementary file 3 — The release of S100A11 protein remains unchanged in PBMCs (A) upon the treatment with pro-inflammatory cytokines, LPS or poly I:C. Synovial fibroblasts up-regulate the release of S100A11 when stimulated with poly I:C (B). Protein levels in cell culture supernatants were measured after 24 h. The horizontal line represents the median. (TIF 85 kb) [file 13075_2017_1288_MOESM3_ESM.tif]
